# Study on the trend of congenital heart disease inpatient costs and its influencing factors in economically underdeveloped areas of China, 2015–2020: a case study of Gansu Province

**DOI:** 10.3389/fpubh.2024.1303515

**Published:** 2024-02-01

**Authors:** Shengze Zhou, Yaya Yang, Lei Wang, Heming Liu, Xuemei Wang, Changping Ouyang, Jinhua Pan, Xiaobin Hu

**Affiliations:** Institute of Epidemiology and Health Statistics, School of Public Health, Lanzhou University, Lanzhou, Gansu, China

**Keywords:** congenital heart disease, inpatient costs, economic burden, influencing factors, random forest

## Abstract

**Background:**

Economic data on congenital heart disease are scarce in economically underdeveloped areas of China. Therefore, this study aimed to shed light on the level and changing trend of congenital heart disease inpatients' economic burden in underdeveloped areas.

**Method:**

This study used a multi-stage stratified cluster sampling method to select 11,055 inpatients with congenital heart disease from 197 medical and health institutions in Gansu Province. Their medical records and expenses were obtained from the Hospital Information System. Univariate analysis was conducted using the rank sum test and Spearman rank correlation. Quantile regression and random forest were used to analyze the influencing factors.

**Results:**

From 2015 to 2020, the average length of stay for congenital heart disease patients in Gansu Province was 10.09 days, with an average inpatient cost of USD 3,274.57. During this period, the average inpatient costs per time increased from USD 3,214.85 to USD 3,403.41, while the average daily inpatient costs increased from USD 330.05 to USD 376.56. The average out-of-pocket costs per time decreased from USD 2,305.96 to USD 754.77. The main factors that affected the inpatient costs included length of stay, cardiac procedure, proportion of medications, age, and hospital level.

**Conclusion:**

Congenital heart disease causes a significant economic burden on both families and society. Therefore, to further reduce the patient's financial burden, the length of stay should be reasonably reduced, and the rational distribution of medical resources should be continuously promoted to ensure equitable access to healthcare services.

## 1 Introduction

Congenital heart disease (CHD) is the most common congenital anomaly worldwide and accounts for nearly one-third of all congenital birth defects (1). The Global Burden of Diseases (GBD) study estimated that 13.3 million people globally lived with CHD, and in 2019 there were 2305.2 congenital heart disease per 100,000 live births (2). Over the past 20 years, the overall mortality of CHD has declined by 60.4%, from 7.1 per 100,000 in 1990 to 2.8 per 100,000 in 2019 (2). However, this rate remains relatively high in low and middle-income countries. In China, the incidence of congenital heart disease has increased by a factor of 4.3, from 3.3 per 1,000 perinatal cases (including stillbirths and live births) in 2010 to 17.3 per 1,000 perinatal cases in 2020 (3). Another large prospective multicenter screening study in China reported 8.98 cases of CHD per 1,000 live births (4). The global healthcare burden of congenital heart disease is increasing, particularly in economically underdeveloped areas. It has become a global and severe public health problem.

Advancements in cardiovascular medicine and surgery for CHD have significantly improved survival rates, enabling nearly most patients to reach adulthood (5–7). However, these medical strides have escalated treatment costs, imposing substantial economic burdens on families affected by CHD and society. A National Health Interview Survey (NHIS) in the US revealed that around half of families with CHD children faced financial challenges due to medical expenses (8). Additionally, analysis of Healthcare Cost and Utilization Project (HCUP) data indicated that CHD patients incurred a total cost of approximately USD 6.1 billion, constituting 26.6% of birth defects-related expenses (9). Despite China currently holding the highest number of CHD patients globally (10, 11), studies on the CHD economic burden, especially in economically underdeveloped areas, are limited. Economically underdeveloped areas are defined by lower levels of economic development, indicated by factors such as low per capita income, high poverty rates, limited access to basic services and infrastructure, high unemployment rates, and a less diversified economy (12).

To grasp the economic burden of congenital heart disease (CHD) in economically underdeveloped regions, we studied Gansu Province in northwestern China—a region with a resident population of 25.01 million and a GDP of 901.67 billion Chinese yuan, ranking fifth lowest in China (13). In Gansu province, urban and rural resident medical insurance has a RMB5000 deductible and a 60% to 80% reimbursement rate (14). The level of care varies among hospital types, with general hospitals providing more comprehensive cardiologist teams and higher-level diagnosis and treatment compared to primary medical institutions. This study aimed to describe the composition and changing trends of CHD inpatient costs within Gansu Province between 2015 and 2020. Additionally, we explored the factors influencing the average inpatient cost per time and daily inpatient cost for patients with CHD and their significance. Based on these results, we discussed potential solutions for reducing the economic burden of CHD and informing future health policy development and practice.

## 2 Methods

### 2.1 Data source

In this study, a multistage stratified cluster random sampling method was adopted. In the first stage, simple random sampling was used to select half of the number of provincial general and Traditional Chinese Medicine hospitals (TCM hospitals) and one of each different type of specialist hospitals. In the second stage, five sample cities were selected, namely Pingliang, Tianshui, Wuwei, Zhangye, and Dingxi, after a full consideration of the economic development, medical conditions, and geographic locations of 14 cities and prefectures within Gansu Province. The same sampling principle used for selecting provincial medical and health institutions was also applied in choosing municipal institutions. In the third stage, one district and two counties, a total of 15 districts (counties), were selected according to the geographical location and urban and rural characteristics of the sample cities, and their medical and health institutions were chosen in the same way as the provincial institutions. In the fourth stage, 5–8 township health centers or community health service centers were randomly selected from the selected districts (counties). Ultimately, a total of 197 medical and health institutions were included. A detailed selection process flowchart was presented in Supplementary Figure S1.

Our research data was collected from the Hospital Information System (HIS) in each sample institution from 2015 to 2020, including general demographic information (age, sex, insurance status, disease diagnosis, time of admission and discharge, length of stay, comorbidity, etc.) and inpatient costs including different cost components. Comorbidities were defined using Elixhauser's translated ICD-10 coding algorithms (15). Comorbidities include cardiovascular disease, neurologic disease, endocrine disease, pulmonary disease, psychiatric disease, or neoplasm (16). ICD codes are listed in Supplementary Table S1.

Based on the International Classification of Diseases 10 (ICD-10) codes Q20–Q26 (17), the medical records of CHD inpatients were extracted from each sample institution. In addition, we defined 12 types of CHD as critical CHD (CCHD) according to the US CDC criteria (18, 19), namely, coarctation of the aorta (COA), double-outlet right ventricle (DORV), d transposition of the great vessels (DTGA), Ebstein malformation, hypoplastic left heart syndrome (HLHS), interrupted aortic arch (IAA), pulmonary valve atresia, single ventricle (SV), total anomalous pulmonary venous return (TAPVR), tetralogy of Fallot (TOF), Tricuspid atresia, and persistent truncus arteriosus (PTA).

### 2.2 Inpatient costs components

Inpatient costs were predefined by the HIS and categorized into seven cost types: treatment costs, medication costs, general medical service fees, examination costs, surgical costs, nursing costs, and other costs. Medication costs included Western pharmaceuticals costs and Traditional Chinese Medicine costs. General medical service fees included beds costs and consultation costs. Examination costs included lab testing costs and medical imaging costs. Other costs included all costs except those mentioned above. The proportion of medications denotes the percentage of medication costs about the total inpatient costs.

### 2.3 Data pre-processing

Before analysis, patient information and medical records underwent anonymization and de-identification. The data were verified for integrity and authenticity to ensure the study's data accuracy. Following logical rectification, non-conforming inpatients were excluded, including (1) primary diagnosis of non-congenital heart disease. (2) Missing major entries and logical errors between costs. (3) Extreme values in inpatient costs. A total of 11,055 medical records of CHD inpatients were eventually collected in our study. To eliminate the interference of price factors on the inpatient costs of CHD patients in different years, the costs were discounted comparably using the Consumer Price Index (CPI) of Gansu Province for 2020 as the base year, and all costs presented in 2020 Chinese Yuan (¥), and they were adjusted when needed. The economic data used in this study, including the CPI, per capita disposable income, and the annual average exchange rate of the Chinese Yuan against the US dollar in 2020 (1:6.8976), were obtained from the National Bureau of Statistics of China.

### 2.4 Statistical analysis

Database building, cleaning and statistical analysis were conducted using Stata 16.0 and R4.2.1 software. Categorical variables were presented as frequency (percentage) [n(q%)] and continuous variables were presented as mean and standard deviation (SD) [mean (SD)]. Since inpatient costs were found be positively skewed, the median and quartiles [M (P25, P75)] was used to describe their distribution, and were examined using a non-parametric test (Mann–Whitney U test and Kruskal–Wallis H test). The correlations of inpatient costs with age, length of stay, proportion of medications, and year was tested using Spearman rank correlation. The items that were statistically significant in the univariate analysis results were set as independent variables. The inpatient costs were taken logarithmically and included as dependent variables in the quantile regression model and the random forest (RF) model to analyze the factors influencing inpatient costs and their importance. Based on previous report (20), the 10th, 50th, and 90th percentiles of inpatient costs were selected for the quantile regression analysis in this study. This provides a comprehensive description of the association between factors influencing and outcomes for low, medium, and high inpatient costs. RF is an ensemble machine learning method developed by Leo Breiman in 2001 (21) that measures the importance of variables better researched than other machine learning methods (22). In the RF model, the 2015–2020 dataset was randomly split into training and testing sets in the ratio of 7:3. And the number of trees to grow (ntree) was kept at 500 by default, and the number of variables used at each split (mtry) was set to 5, which was demonstrated to be able to lead to a much more stable variable importance estimate (23). Variable assignment is shown in Supplementary Table S2 and *P* values < 0.05 were considered statistically significant.

## 3 Results

### 3.1 Demographic characteristics and univariate analysis

A total of 11,055 congenital heart disease hospitalizations were identified and analyzed in this study from 2015 to 2020, including 5,841 males (52.84%) and 5,214 females (47.16%). The average age was 29.38 ± 25.34 years old. Of them, 4,870 (44.05%) were children under the age of 18. Supplementary Table S3 presented the basic characteristics of patients in different age groups. The average length of stay was 10.09 ± 9.16 days. Out-of-pocket was listed as the primary payment method for most admissions (52.84%). The overall reimbursement rate of inpatient costs for patients with CHD was 58.93%. In terms of hospital level (graded by administrative subordination), almost 80% of CHD inpatients were from provincial or municipal medical and health institutions. From the included admissions, over 70% (73.12%) were treated in general hospitals, while < 2% were seen in primary medical and health institutions. Nearly half of (46.49%) the patients underwent cardiac surgery, and the cost of hospitalization was significantly higher than for non-surgical patients. Basic characteristics of cardiac procedure patients and non-cardiac procedure patients are presented in Supplementary Table S4. Among inpatients with different types of congenital heart disease, almost half of the inpatients (49.26%) were congenital malformations of cardiac septa. The proportion of CCHD inpatients was lower at 3.5% compared to those with non-severe CHD. Supplementary Table S5 provided more details on the characteristics of patients with CCHD vs. non-severe cases. Inpatient costs and ICD-10 codes for CHD types and subtypes in Gansu Province from 2015 to 2020 were presented in Supplementary Table S6.

Univariate analysis showed statistically significant differences in the costs of CHD patients per hospitalization in terms of age, payment method, hospital nature, hospital level, hospital type, number of comorbidities, length of stay, the proportion of medications, cardiac procedure, and CHD severity (*P* < 0.001), was presented in Table 1.

**Table 1:** Basic characteristics of patients and univariate analysis of average hospitalization costs per time.

**Category**	**n(q%)**	**Median (lower quartile, upper quartile) (CNY)**	**Test statistic**	***P* value**
All cases	11,055	22,586.68 (4,712.93, 3,2071.16)	-	*-*
Gender	-	-	1.836	0.066
Male	5,841 (52.84)	13,085.44 (4,799.57, 32,400.20)	-	-
Female	5,214 (47.16)	11,327.64 (4,596.44, 31,542.08)	-	-
Age(years) [mean (SD)]	29.38 (25.34)	-	−0.132	< 0.001
< 1	1,236 (11.18)	4,167.68 (1,092.44, 35,335.33)	-	-
1–4	2,162 (19.56)	31,475.51 (9,095.04, 46,142.56)	-	-
5–17	1,472 (13.32)	27,931.73 (16,724.35, 35,974.88)	-	-
18–64	5,242 (47.42)	8,785.79 (4,842.62, 23,789.26)	-	-
≥65	943 (8.53)	7,231.37 (4,630.49, 10,952.15)	-	-
Payment method	-	-	−19.585	< 0.001
Medical insurance reimbursement	5,214 (47.16)	7,958.29 (4,066.82, 25,333.94)	-	-
Out-of-pocket	5,841 (52.84)	19,367.66 (6,239.50, 36,526.54)	-	-
Reimbursement rate (%) [mean (SD)]	58.93 (46.83)	-	−0.0203	< 0.05
Hospital nature	-	-	5.304	< 0.001
Public	10,960 (99.14)	12,234.27 (4,724.78, 32,195.96)	-	-
Private	95 (0.86)	6,260.06 (3,711.40, 10,291.76)	-	-
Hospital level	-	-	34.328	< 0.001
Provincial and municipal level	8,818 (79.76)	18,491.46 (6,528.77, 35,204.50)	-	-
District and county level and below	2,237 (20.24)	4,413.99 (2,844.69, 8,512.31)	-	-
Hospital type	-	-	549.732	< 0.001
General hospital	8,083 (73.12)	11,759.69 (5,378.23, 29,113.54)	-	-
Traditional Chinese Medicine (TCM) hospital	505 (4.57)	3,693.59 (2,465.12, 6,001.73)	-	-
Maternal and Child Healthcare Hospital	1,937 (17.52)	28,692.19 (3,255.72, 50,527.40)	-	-
Specialized hospital	320 (2.89)	15,204.52 (4,826.21, 35,822.28)	-	-
Primary medical and health institution^a^	210 (1.90)	23,944.33 (6,847.859, 44,275.97)	-	-
Length of stay (days) [mean (SD)]	10.09 (9.16)	-	0.638	< 0.001
Proportion of medications (%) [mean (SD)]	15.74 (15.25)	-	−0.118	< 0.001
Number of comorbidities^b^	-	-	115.5	< 0.001
0	5,438 (49.19)	17,307.30 (4,564.88, 33,972.08)	-	-
1	1,932 (17.48)	17,640.44 (6,186.01, 31,731.41)	-	-
2	1,737 (15.71)	7,961.57 (4,091.68, 22,756.40)	-	-
≥3	1,948 (17.62)	8,651.23 (4,674.48, 31,885.36)	-	-
Cardiac procedure	-	-	−63.883	< 0.001
No	5,916 (53.51)	5,885.11 (2,955.57, 11,589.50)	-	-
Yes	5,139 (46.49)	31,071.38 (16,827.06, 45,325.44)	-	-
CHD severity	-	-	−9.357	< 0.001
Critical congenital heart disease(CCHD)	387 (3.50)	32,838.06 (5,689.15, 73,673.41)	-	-
Non-severe CHD	10,668 (96.50)	11,822.00 (4,678.95, 33,1162.97)	-	-

^a^Primary medical and health institutions include township health centers and community health service centers. ^b^Comorbidities were defined using the Elixhauser's translated ICD-10 coding algorithms. These include conditions related to the cardiovascular system and other systems.

### 3.2 Component of inpatient costs for CHD

**Figure 1:**
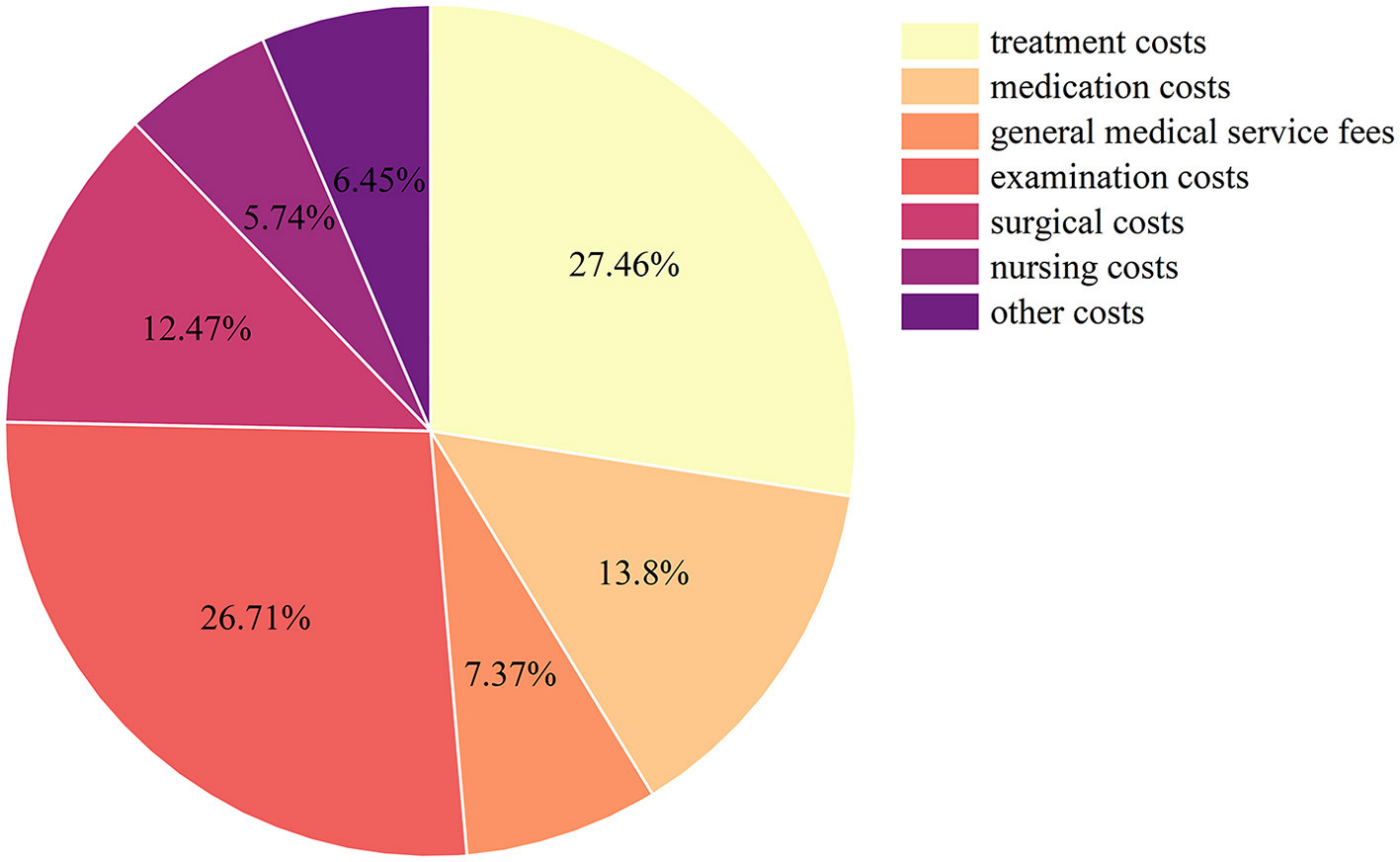
Components of inpatient costs of CHD patients in Gansu Province, China, from 2015 to 2020.

From 2015 to 2020, the average inpatient cost per hospitalization for CHD patients in Gansu Province amounted to CNY 22,586.68 (USD 3,274.57). To dissect the expenditure in detail, the treatment costs of CNY 6,200.17 (USD 898.89, 27.45%) and the examination costs of CNY 6,030.72 (USD 874.32, 26.7%), which were the major component of inpatient costs. They were followed by the medication costs of CNY 3,117.33 (USD 451.94, 13.80%), the surgical costs of CNY 2,816.87 (USD408.38, 12.47%), and the general medical service fees of CNY 1,665.17 (USD 241.41, 7.37%). The lowest percentage was attributed to nursing costs at CNY 1,297.58 (USD 188.12, 5.74%), as shown in Figure 1.

### 3.3 The trend of inpatient costs for CHD

As shown in Table 2, the per hospitalization costs for CHD patients in Gansu Province showed a general upward trend from CNY 22,174.72 (USD 3,214.85) in 2015 to CNY 23,475.41 (USD 3,403.41) in 2020. The average growth rate over the 6-year period was 1.15%, although this change did not reach statistically significant (*P* = 0.2508). Meanwhile, the average daily inpatient costs for CHD followed a similar trend, rising from CNY 2,276.58 (USD 330.05) to CNY 2,597.35 (USD 376.56, *P* < 0.001), during the same period, with an average growth rate of 2.67%. In addition, we found that average inpatient out-of-pocket costs increased from 2015 to 2017 but declined between 2018 and 2020 (*P* < 0.001), and the average growth rate was −20.02%.

**Table 2:** Trends of inpatient costs for CHD.

**Year**	**Inpatient costs per time (CNY)^a^**	**Growth rate (%)^b^**	**Out-of-pocket costs (CNY)^a^**	**Growth rate (%)**	**Daily inpatient costs (CNY)**	**Growth rate (%)**
2015	22,174.72	-	15,905.58	-	2,276.58	-
2016	22,494.61	1.44^b^	18,579.07	16.81	2,543.31	11.72
2017	20,881.67	−7.17	19,409.82	4.47	1,853.89	−27.11
2018	22,280.57	6.70	18,655.53	−3.89	2,187.84	18.01
2019	23,765.66	6.67	7,645.67	−59.02	2,338.82	6.90
2020	23,475.41	−1.22	5,206.07	−31.91	2,597.35	11.05
Overall^c^	22,586.68	1.15^c^	13,817.94	−20.02	2,291.25	2.67
***P*** **value**	0.251	-	< 0.001	-	< 0.001	-

^a^The mentioned costs are the costs of CHD patients per hospitalization. ^b^The annual growth rate is the year-over-year growth rate based on the previous year. ^c^The overall growth rate is the average growth rate from 2015 to 2020.

### 3.4 Quantile regression analysis of inpatient costs

The results from the quantile regression analysis revealed that age, payment method, hospital level, length of stay, proportion of medications, and cardiac procedure significantly influenced all three percentile levels of inpatient costs per time and average daily inpatient costs (all *P* < 0.001). The quantile regression Pseudo R^2^ in the 10th, 50th, and 90th percentile of inpatient costs per time was 0.356, 0.422, and 0.399, compared with 0.242, 0.296, and 0.199 for average daily inpatient costs.

Hospital level and proportion of medications had a significant negative impact (*P* < 0.001) on all percentile points of inpatient costs per time and average daily inpatient costs for CHD patients. The intensity of this effect was higher in the middle and high quartiles than in the low quartiles. Age and payment method had a positively significant impact (*P* < 0.001) on all percentile levels of inpatient costs per time and average daily inpatient costs. For average daily inpatient costs, patients treated in private hospitals had lower costs at the 90th percentile and higher costs at the 10th percentile compared to public hospitals (*P* < 0.05). Among hospital types, inpatient costs were lower on all percentile levels for patients treated in TCM hospitals than in general hospitals. In contrast, patients treated in specialized hospitals and primary medical and health institutions had higher inpatient costs at both the 50th and 90th percentiles than those treated in general hospitals (*P* < 0.05). For the number of comorbidities, inpatient costs per time and average daily inpatient costs were higher at the 10th percentile and lower at the 90th percentile for patients with comorbidities in comparison to those without comorbidities (all *P* < 0.05). Length of stay had a significant positive effect on all percentiles of inpatient costs per time. However, for average daily inpatient costs, length of stay had a positive effect at the lower percentile and negative impacts at the remaining two percentiles (*P* < 0.001). Cardiac procedure had a significant positive effect on inpatient costs at all percentile levels (*P* < 0.001). Patients with CCHD had higher average daily inpatient costs on all percentile levels compared to non-severe CHD patients (*P* < 0.05). However, this finding was not statistically significant for inpatient costs per time, as shown in Table 3.

**Table 3:** Quantile regression analysis of CHD inpatient costs (CNY).

**Variables**	**Inpatient costs per time**			**Average daily hospitalization costs**		
	**Q10**	**Q50**	**90**	**Q10**	**Q50**	**90**
Age	0.014^†^	0.004^†^	0.004^†^	0.009^†^	0.005^†^	0.005^†^
**Payment method (contrast** = **medical insurance reimbursement)**						
Out-of-pocket	0.149^†^	0.231^†^	0.137^†^	0.173^†^	0.236^†^	0.183^†^
**Hospital nature (contrast** = **public)**						
Private	0.178^*^	−0.408^*^	−0.340^†^	0.235^*^	−0.127	−0.418^†^
**Hospital level (contrast** = **provincial and municipal level)**						
District and county level and below	−0.563^†^	−0.657^†^	−0.706^†^	−0.536^†^	−0.603^†^	−0.597^†^
**Hospital type (contrast** = **general hospital)**						
Traditional Chinese Medicine (TCM) hospital	−0.190^*^	−0.179^†^	−0.261^*^	−0.165^*^	−0.237^†^	−0.270^†^
Maternal and Child Healthcare Hospital	−0.231^†^	−0.176^†^	0.199^†^	0.054	0.043^*^	0.287^†^
Specialized hospital	−0.173	0.686^†^	1.679^†^	−0.226^*^	0.552^†^	1.509^†^
Primary medical and health institution	0.283	1.109^†^	1.700^†^	0.116	1.072^†^	1.677^†^
**Number of comorbidities (contrast** = **0)**						
1	0.343^†^	−0.068	−0.059^*^	0.266^†^	−0.094^*^	−0.140^†^
2	0.251^†^	−0.107^†^	−0.202^†^	0.176^†^	−0.134^†^	−0.257^†^
≥3	0.179^*^	−0.063	−0.102^*^	0.097^*^	−0.063^*^	−0.173^†^
Length of stay	0.068^†^	0.062^†^	0.057^†^	0.007^†^	−0.005^†^	−0.010^†^
Proportion of medications	−0.007^†^	−0.010^†^	−0.014^†^	−0.013^†^	−0.17^†^	−0.019^†^
**Cardiac procedure (contrast** = **No)**						
Yes	0.938^†^	0.996^†^	0.630^†^	0.853^†^	0.745^†^	0.372^†^
**CHD severity (contrast** =**non-severe CHD)**						
CCHD	0.125	0.040	0.065	0.205^†^	0.153^†^	0.198^†^
**Year (contrast** = **2015)**						
2016	0.115^*^	−0.010	0.081^*^	0.075	−0.033	−0.010
2017	−0.048	−0.091^*^	−0.015	−0.045	−0.090^†^	0.005
2018	−0.315^†^	−0.051^*^	0.017	−0.236^†^	−0.104^*^	0.000
2019	−0.461^†^	0.005	0.116^†^	−0.293^†^	−0.001	0.129^*^
2020	−0.105	0.126^†^	0.319^†^	−0.002	0.184^†^	0.310^†^
Pseudo ***R**^**2**^*	0.356	0.422	0.399	0.242	0.296	0.199

^*^P < 0.05, ^†^*P* < 0.001.

### 3.5 Random forest analysis of inpatient costs

For inpatient costs per time, the root mean square error (RMSE) of the RF model was 0.266 with *R*^2^ = 0.830, and for average daily inpatient costs, the RMSE was 0.264 with *R*^2^ = 0.703.

**Figure 2:**
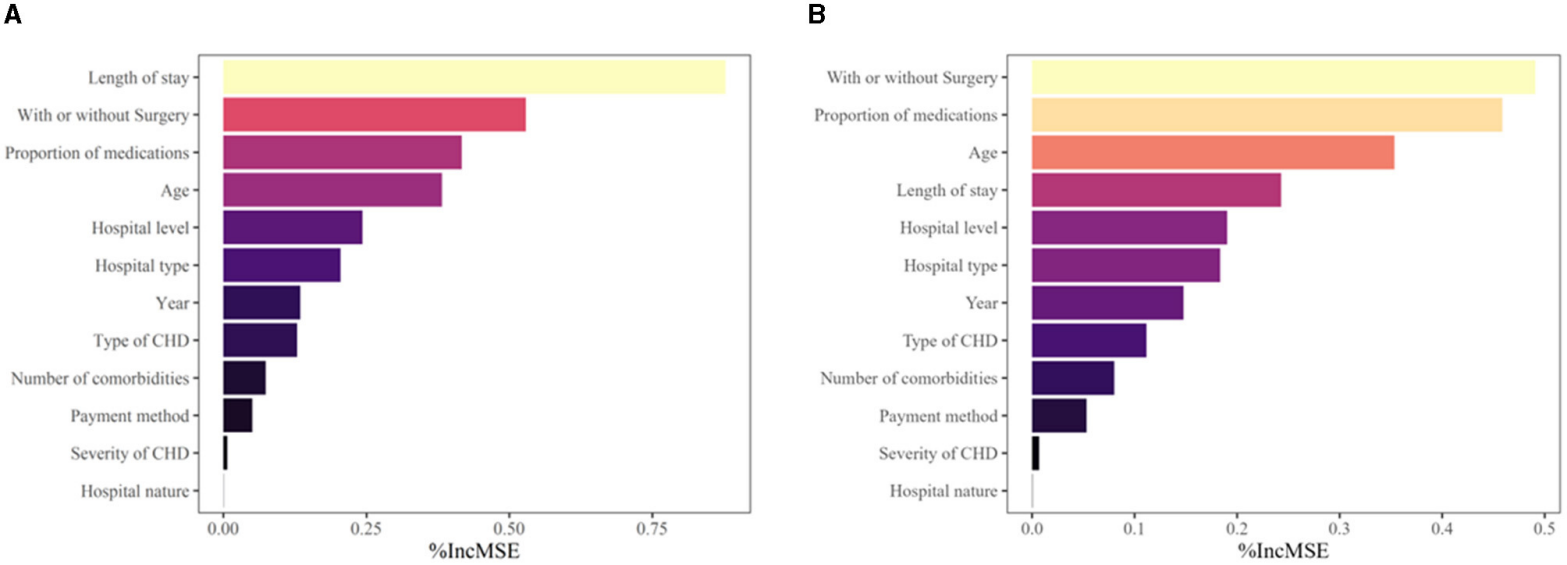
Analysis of the importance of factors influencing inpatient costs for patients with CHD. **(A)** Ranking of the importance of factors influencing inpatient costs per time for patients with CHD. **(B)** Ranking of the importance of factors influencing daily inpatient costs for patients with CHD.

Figure 2 shows the factors influencing inpatient costs in descending order of importance according to the average precision of decline (%IncMSE). The results showed that the top five influencing factors for inpatient costs per time were length of stay, cardiac procedure, proportion of medications, age, and hospital level. The top five factors influencing average daily inpatient costs were cardiac procedure, proportion of medications, age, length of stay, and hospital level.

## 4 Discussion

This study is the first to systemically report the trends and factors influencing inpatient costs of CHD patients in economically underdeveloped areas of northwestern China, thus providing substantial insights into the economic burden of congenital heart disease in China. We found that adults over the age of 18 between 2015 and 2020 accounted for the largest patient group, but infants younger than 1 year had the highest inpatient costs. This may be attributed to advancements in diagnosis and treatment during infancy and childhood, resulting in higher survival rates for patients with CHD (24, 25). Consequently, the increasing number of adults with congenital heart disease (ACHD), outnumber the population of children living with CHD (26). Additionally, this may also be related to the delayed healthcare-seeking behavior of congenital heart disease patients due to medical resources and lower educational levels in Gansu Province. Our study revealed that the average length of stay was 10.09 ± 9.16 days, which was longer than those in other reports (27, 28). It is worth noting that almost half of the patients in this study underwent cardiac procedures, which could have contributed to their prolonged length of stay. A previous study has indicated that patients with congenital heart disease require intensive care unit (ICU) admission post-surgery, which further extends their length of stay, and it is important to consider that the longer the ICU stay, the more healthcare resources and associated costs are utilized (29). In our study, the observed proportion of patients with CCHD was comparatively lower than reported in other studies (17, 30). This discrepancy may be associated with the constrained medical resources and inadequate advanced diagnostic and therapeutic capabilities within Gansu Province. Under such circumstances, patients with CCHD may demonstrate a tendency to seek medical attention in provinces with more sophisticated medical facilities. Moreover, our study has observed higher inpatient costs for patients with CCHD and congenital heart surgery. This is consistent with the findings of other similar CHD studies (28, 30, 31).

In our study, the average inpatient costs per time of CHD patients in Gansu Province from 2015 to 2020 was CNY 22,586.68 (USD 3,274.57), which was higher compared with a similar study conducted on adults with congenital heart disease in Hong Kong, China (32). In contrast, inpatient costs in Gansu Province were significantly lower than the findings of the National Center for Cardiovascular Disease (33). Moreover, compared to other high-income countries the inpatient costs of congenital heart disease are significantly lower in Gansu Province. For instance, a study from Canada revealed that for children in their 1^st^ year of life, the median (IQR) cost per child, excluding drug costs, was $224,324 ($127,596–$312,077) (34). A study conducted in Arkansas found that the median per-patient hospital costs for all hospitalizations were $28,482 (interquartile range [IQR]: $10,899, $53,530) (26). Another study on the costs of congenital heart defects in American adolescents showed median (IQR) costs and costs per admission of $27,304 ($47,448) and $10,720 ($18,246), respectively (24). The difference in cost between our study with others might be related to various factors, such as the year of the study, the economic level of the countries, the method of treatment utilized, and the criteria for inclusion and exclusion. Additionally, it is worth noting that the use of ICD-9 codes in US studies and ICD-10 codes in ours could also play a role in the differences observed. Despite the lower inpatient costs observed in Gansu Province compared to developed countries, compared to the national economic data, the total expenditures exceed the per capita disposable income of CNY 20,335 in the province in 2020, indicating a significant financial burden for CHD patients and their families.

Treatment, examination, medication, and surgery accounted for a high proportion of inpatient costs, with these four items accounting for more than 80% of the total costs, similar to other reports (32, 35). The examination costs are considerably high, possibly attributed to using costly technologies such as color Doppler ultrasound, spiral CT, magnetic resonance imaging, and cardiac catheterization to diagnose CHD. In Gansu Province, the average inpatient costs per time and daily inpatient costs of CHD patients showed a generally rising trend from 2015 to 2020. However, an abnormal downward trend was observed in 2017, which could be attributed to the zero-markup policy (ZMP) for essential medicines in Gansu Province. This policy eliminated the 15% profit margin previously allowed for drug sales at public hospitals. A previous study (36) on the impacts of ZMP on medical expenditures found that the policy led to a short-term decrease in total inpatient costs and medicine expenses per patient in pilot hospitals. However, the difference disappeared over the long term. Moreover, we found that out-of-pocket costs per time for patients with CHD decreased after 2017, with an average growth rate of −20.02%. This trend seems related to the continuous improvement of the national health insurance system and the gradual increase of reimbursement rates in recent years.

The results of the quantile regression analysis and the random forest regression tree model indicated that among the variables considered, the length of stay, cardiac procedure, the proportion of medications, age, and hospital level were the main factors influencing inpatient costs. Prior studies (28, 37, 38) have established that length of stay, surgical procedures, and age play a significant role in determining the cost of hospitalization. Our results add to the literature by showing that medication proportion and hospital level were critical factors impacting inpatient costs. As such, interventions targeting these variables should be prioritized to reduce the economic burden of cardiac disease. Specifically, efforts to decrease the length of stay and optimize medication proportion likely yield the most significant impact. Children, especially infants, had significantly higher inpatient costs. Therefore, more attention should be paid to this population, and a multidisciplinary program is needed to obtain better outcomes. Hospital level as a significant influence on inpatient costs also corresponds to the “Two-Eight Rule” by the 2021 Medical Quality Report of Cardiovascular Diseases in China (39), meaning that 20% of the large provincial and municipal hospitals implement 80% of the treatment. This principle results in the regionalization of medical resources. Medical institutions that offer higher levels of medical specialization and better equipment tend to charge more for their services. This is particularly evident in provincial and municipal institutions, where a higher percentage of patients (89.96%) undergo cardiac surgery due to their advanced medical capabilities and specialized services. As a result, patients in such institutions may face higher medical costs. Consequently, it is necessary to promote the equitable allocation of medical resources across different areas and strengthen the development of primary medical institutions to address this issue.

Several limitations should be addressed for this study. First, only the inpatient costs of patients with the first diagnosis of CHD were analyzed. The costs of patients with CHD who were admitted for other reasons, as well as indirect costs, were not included. Second, the data for the study came from the hospital information system. Thus, the variables included have limitations and cannot include other variables, such as the type of admission that may affect the cost of hospitalization, and the purpose of the indexed hospitalization of the included patients. Third, we have not adequately considered the potential impact of major changes in policies on the trend of inpatient costs. Lastly, our study only reflected the costs of patients with CHD in Northwest China. However, special attention should be given to the fact that this study represents the first systematic report on trends and influencing factors related to inpatient costs for CHD patients in China. Therefore, our findings might help further understand the financial burden caused by congenital heart disease surgery and facilitate the adjustment of health insurance policies.

## 5 Conclusion

This is the first report on the changing trend and influencing factors study of CHD patients in economically underdeveloped areas of northwestern China. Our study has identified that the length of stay, cardiac procedure, the proportion of medications, age, and hospital level were the main factors influencing inpatient costs. Furthermore, it showed that CHD causes a significant economic burden on both families and society. Therefore, to further reduce the patient's financial burden, the length of stay should be reasonably reduced, and the rational distribution of medical resources should be continuously promoted to ensure equitable access to healthcare services.

## Data availability statement

The raw data supporting the conclusions of this article will be made available by the authors, without undue reservation.

## Author contributions

SZ: Data curation, Methodology, Writing – original draft, Investigation. YY: Data curation, Investigation, Validation, Writing – review & editing. LW: Writing – review & editing, Data curation, Investigation, Validation. HL: Data curation, Investigation, Validation, Writing – review & editing. XW: Data curation, Investigation, Validation, Writing – review & editing. CO: Data curation, Investigation, Validation, Writing – review & editing. JP: Data curation, Investigation, Validation, Writing – review & editing. XH: Resources, Supervision, Writing – review & editing.
