# Supplementary material for: Study on the trend of congenital heart disease inpatient costs and its influencing factors in economically underdeveloped areas of China, 2015–2020: a case study of Gansu Province
Source: Front Public Health. 2024 Feb 1;12:1303515. doi: 10.3389/fpubh.2024.1303515 (PMC10867958; doi:10.3389/fpubh.2024.1303515)
Supplement: Supplementary file 1 [file Data_Sheet_1.pdf]

Supplementary Material

1 Supplementary Tables

Supplemental Table S1: ICD-10 codes for CHD comorbidities.

Comorbidities	ICD-10
Cardiovascular disease	I00-I99
Neurological disease	G00-G99
Endocrine disease	E00-E90
Pulmonary disease	J00-J99 (except J00-06 & J30-39)
Psychiatric disease	F00-F99
Neoplasm	C00-D48

Supplemental Table S2: Variable assignment of quantile regression and random forest model.

Variables	Variable assignment
Age(years)	Actual value
Payment method	1 = Medical insurance reimbursement, 2 = Out-of-pocket
Hospital nature	1 = Public, 2 = Private
Hospital level	1 = Provincial and municipal level,<br>2 = District and county level, and below
Hospital type	1 = General hospital, 2 = TCM hospital,<br>3 =Maternal and Child Healthcare Hospital,<br>4 = Specialized hospital, 5 = Primary medical and health institution
Length of stay (days)	Actual value
Proportion of medications (%)	Actual value
Number of comorbidities	0 = 0, 1 = 1, 2 = 2, 3 = 3 or more
Cardiac procedure	0 = No, 1 = Yes
CHD severity	1 = Non-severe CHD, 2= CCHD
Year[a]	1 = 2015, 2 = 2016, 3 = 2017, 4 = 2018, 5 = 2019, 6 = 2020
Inpatient costs per time	Ln (actual value)
Average daily inpatient costs	Ln (actual value)

[a]The year is based on the patient’s discharge time in the hospital information system.

Supplemental Table S3: Basic characteristics of patients in different age groups.

Category	<1		1-4		5-17		18-64		≥65	
	n(q%)	Median (lower quartile, upper quartile) (CNY)	n(q%)	Median (lower quartile, upper quartile) (CNY)	n(q%)	Median (lower quartile, upper quartile) (CNY)	n(q%)	Median (lower quartile, upper quartile) (CNY)	n(q%)	Median (lower quartile, upper quartile) (CNY)
All cases	1236(11.18)	4167.68(1092.44,35335.33)	2162(19.56)	31475.51(9095.04,46142.56)	1472(13.32)	27931.73(16724.35,35974.88)	5242(47.42)	8785.79(4842.62,23789.26)	943(8.53)	7231.37(4630.49,10952.15)
Gender										
Male	587(47.49)	4186.25(1154.91,36837.50)	1059(48.98)	32154.30(11749.28,45488.18)	790(53.67)	28245.45(16818.74,36202.65)	2889(55.11)	9236.63(4865.12,24868.82)	516(54.72)	7011.39(4574.46,10446.16)
Female	649(52.51)	4145.76(1023.59,33903.31)	1103(51.02)	30961.69(7389.46,47308.29)	682(46.33)	27667.57(16624.81,35497.22)	2353(44.89)	8394.14(4796.09,21511.89)	427(45.28)	7418.56(4799.72,11251.40)
Payment method										
Medical insurance reimbursement	476(38.51)	7058.25(1001.75,39951.35)	646(29.88)	32601.71(14483.63,43574.57)	375(25.48)	23996.76(5953.24,33995.46)	3044(58.07)	7092.95(3981.10,18976.48)	673(71.37)	6567.54(4464.05,9535.34)
Out-of-pocket	760(61.49)	3189.68(1131.35,25146.48)	1516(70.12)	30946.00(7200.21,47593.00)	1097(74.52)	28914.76(17960.13,36427.85)	2198(41.93)	15274.95(7054.70,28104.26)	270(28.63)	9050.43(5817.88,18767.47)
Hospital nature										
Public	1222(98.87)	4029.98(1084.34,35818.20)	2147(99.31)	31764.94(9691.52,46331.26)	1466(99.59)	28034.48(16780.82,35992.42)	5194(99.08)	8826.69(4865.16,23890.01)	931(98.73)	7231.37(4628.25,10952.15)
Private	14(1.13)	8648.20(5730.96,13111.89)	15(0.69)	5722.17(2765.79,15087.87)	6(0.41)	6653.16(4505.53,10291.76)	48(0.92)	5798.69(3475.00,7895.79)	12(1.27)	7491.24(6355.04,11313.85)
Hospital level										

Provincial and municipal level	1125(91.02)	4385.69(1023.68,38292.13)	1989(92.00)	32148.77(11844.09,46996.06)	1330(90.35)	28766.36(18207.04,36501.87)	3759(71.71)	12150.88(6838.19,26909.78)	615(65.22)	8370.63(6254.63,14224.06)
District and county level and below	111(8.98)	3642.04(2345.76,8677.71)	173(8.00)	13810.21(2812.20,36681.87)	142(9.65)	4924.96(1564.22,25248.21)	1483(28.29)	4259.38(2831.21,7634.01)	328(34.78)	4574.46(3346.03,6647.14)
Hospital type										
General hospital	497(40.21)	986.86(843.22,4370.342)	1083(50.09)	30054.97(15258.98,40501.77)	1235(83.90)	27696.27(17054.58,34694.59)	4445(84.80)	9366.32(5480.91,24072.40)	823(87.27)	7503.72(4974.57,11400.43)
Traditional Chinese Medicine (TCM) hospital	18(1.46)	1476.60(1210.29,2981.78)	10(0.46)	2782.45(1758.96,3701.17)	30(2.04)	1934.08(1323.61,3933.79)	370(7.06)	3699.29(2552.66,6121.78)	77(8.17)	4630.49(3450.61,7544.19)
Maternal and Child Healthcare Hospital	694(56.15)	14195.14(2479.81,50632.40)	967(44.73)	35799.77(3470.24,52636.40)	145(9.85)	39543.05(22711.12,50012.00)	129(2.46)	6482.73(3448.33,20310.17)	2(0.21)	29169.48
Specialized hospital	14(1.13)	18105.27(5159.64,55120.46)	53(2.45)	35474.36(17796.67,48427.78)	36(2.45)	22094.54(8882.51,36956.86)	187(3.57)	9863.78(4155.13,29908.04)	30(3.18)	4931.12(3189.91,6962.00)
Primary medical and health institution	13(1.05)	27713.70(10640.13,40281.75)	49(2.27)	35496.45(16311.62,48427.78)	26(1.76)	25900.86(14551.76,39604.84)	111(2.12)	24188.08(5080.59,45334.97)	11(1.17)	4807.54(1036.91,10958.43)
Length of stay (days) [mean(SD)]	10.16(12.30)		13.63(12.46)		10.57(7.94)		8.67(6.86)		8.99(5.28)	
Proportion of medications (%) [mean(SD)]	12.85(12.02)		10.95(10.84)		8.77(9.27)		18.68(16.79)		25.02(17.10)	
Number of comorbidities										
0	572(46.28)	1125.10(855.92,5827.00)	1252(57.91)	33093.05(11038.39,44739.29)	847(57.54)	27860.58(16810.92,36369.44)	2387(45.54)	11261.37(5294.75,25491.30)	380(40.30)	7642.32(4636.70,13315.23)

1	123(9.95)	4449.01(1739.55,39088.01)	414(19.15)	23804.36(3039.12,36111.98)	433(29.42)	28478.20(17194.23,33504.55)	864(16.48)	9933.71(6236.73,28309.59)	98(10.39)	8504.03(6408.31,14284.10)
2	169(13.67)	12095.17(3525.26,54731.89)	215(9.94)	24421.88(5741.97,42996.57)	118(8.02)	23615.24(15575.21,31454.43)	1018(19.42)	7105.01(3969.76,15858.19)	217(23.01)	5878.74(3853.41,8593.31)
≥3	372(30.10)	12293.20(3233.17,49777.58)	281(13.00)	45409.15(21258.02,74064.03)	74(5.03)	34153.83(5170.07,50012.00)	973(18.56)	7192.70(4288.43,16704.60)	248(26.30)	6946.24(4973.70,9915.28)
Cardiac procedure										
No	842(68.12)	1596.96(912.56,5047.86)	564(26.09)	3181.91(1423.96,9822.07)	383(26.02)	8055.26(2316.06,22186.43)	3398(64.82)	6986.96(3963.78,14570.30)	729(77.31)	6352.54(4390.72,9573.22)
Yes	394(31.88)	47497.22(34646.23,71096.6)	1598(73.91)	36999.75(25547.02,52522.49)	1089(73.98)	30739.77(23442.35,38630.45)	1844(35.18)	23355.36(8118.99,39241.55)	214(22.69)	9803.69(7068.00,23169.79)
CHD severity										
CCHD	91(7.36)	21354.00(5471.69,75078.60)	179(8.28)	53947.80(9440.23,85607.15)	65(4.42)	34324.41(3923.06,65078.17)	50(0.95)	7927.16(5000.42,14573.96)	2(0.21)	6206.16
Nonsevere CHD	1145(92.64)	3530.84(1036.00,29660.80)	1983(91.72)	30829.91(9052.86,43976.17)	1407(95.58)	27794.09(16818.74,35466.54)	5192(99.05)	8792.30(4842.59,23840.39)	941(99.79)	7243.11(4630.49,10952.15)

Supplemental Table S4: Basic characteristics of cardiac procedure patients and non-cardiac procedure patients.

Category	Cardiac procedure patients		Non-cardiac procedure patients	
	n(q%)	Median (lower quartile, upper quartile) (CNY)	n(q%)	Median (lower quartile, upper quartile) (CNY)
All cases	5139(46.49)	31071.38(16827.06,45325.44)	5916(53.51)	5885.11(2955.57,11589.50)
Gender				
Male	2774(53.98)	29973.70(16084.25,42320.60)	3067(51.84)	5645.87(2874.43,11509.62)
Female	2365(46.02)	29855.80(16015.62,44942.90)	2849(48.16)	5588.48(2755.15,10559.10)
Age(years) [mean (SD)]	20.82(22.54)		36.82(25.29)	
<1	394(7.67)	45501.74(33801.30,69758.60)	842(14.23)	1534.52(880.18,4828.18)
1-4	1598(31.10)	34982.95(24523.80,50432.00)	564(9.53)	3055.25(1362.76,9089.24)
5-17	1089(21.19)	29722.30(22017.08,36873.40)	383(6.47)	7715.76(2209.57,21028.60)
18-64	1844(35.88)	22098.36(7850.21,37350.40)	3398(57.44)	6738.01(3802.98,13960.54)
≥65	214(4.16)	9290.45(6941.43,21758.30)	729(12.32)	6139.31(4178.04,9192.43)
Payment method				
Medical insurance reimbursement	2130(41.45)	24429.58(8884.52,37658.73)	3084(52.13)	4937.51(2976.68,8434.07)
Out-of-pocket	3009(58.55)	32564.40(20081.30,47205.60)	2832(47.87)	6904.92(2477.74,17920.96)
Hospital nature				
Public	5119(99.61)	29972.30(16146.76,43596.10)	5841(98.73)	5623.03(2803.83,11089.35)
Private	20(0.39)	8818.10(7640.77,28136.14)	75(1.27)	5365.63(3326.58,8104.08)
Hospital level				
Provincial and municipal level	4623(89.96)	31451.79(19472.40,45160.70)	4195(70.91)	6807.72(3219.29,13012.26)
District and county level and below	516(10.04)	6985.27(3760.28,18552.00)	1721(29.09)	3778.42(2510.61,6461.68)
Hospital type				
General hospital	3635(70.73)	27897.80(15120.30,37871.40)	4448(75.19)	6287.18(3380.38,11601.15)
Traditional Chinese Medicine (TCM) hospital	66(1.28)	17175.95(3642.24,22280.11)	439(7.42)	3330.60(2190.05,4711.66)
Maternal and Child Healthcare Hospital	1262(24.56)	41748.30(27404.20,60509.10)	675(11.41)	2183.28(1279.57,6099.45)
Specialized hospital	106(2.62)	18828.70(6817.59,30259.60)	214(3.62)	10531.50(3917.12,33677.50)
Primary medical and health institution	70(1.36)	20037.50(7175.72,38770.40)	140(2.37)	22556.55(5310.98,41506.10)
Length of stay (days) [mean (SD)]	13.54(11.07)		7.09(5.57)	
Proportion of medications (%) [mean (SD)]	12.66(9.70)		18.41(18.37)	
Number of comorbidities				
0	2587(50.34)	30534.90(18237.19,40554.90)	2851(48.19)	5415.03(2165.35,16683.00)

1	1035(20.14)	29688.80(18289.00,41476.50)	897(15.16)	6670.76(3090.63,11183.80)
2	695(13.52)	24479.90(8801.68,8801.68)	1042(17.61)	5243.56(3282.41,8507.48)
≥3	822(16.00)	35850.60(8729.07,64036.46)	1126(19.03)	5734.74(3541.14,10142.40)
CHD severity				
Critical congenital heart disease (CCHD)	249(4.85)	62486.70(33554.85,93029.30)	138(2.33)	4872.41(2671.07,9171.57)
Non-severe CHD	4890(95.15)	29489.95(15766.00,41704.07)	5778(97.67)	5636.09(2811.41,11130.65)

Supplemental Table S5: Basic characteristics of CCHD patients and Non-severe CHD patients.

Category	Critical congenital heart disease (CCHD)		Non-severe CHD	
	n(q%)	Median (lower quartile, upper quartile) (CNY)	n(q%)	Median (lower quartile, upper quartile) (CNY)
All cases	387(3.50)	32838.06(5689.15,73673.41)	10668(96.50)	11822.00(4678.95,331162.97)
Gender				
Male	180(46.51)	33288.89(5509.74,76640.37)	5661(53.07)	12823.44(4779.85,31851.23)
Female	207(53.49)	32838.06(6502.53,72302.61)	5007(46.93)	11072.10(4536.25,30713.31)
Age(years) [mean(SD)]	8.07(14.30)		30.16(25.32)	
<1	91(23.51)	21354.00(5471.69,75078.60)	1145(10.73)	3530.84(1036.00,29660.80)
1-4	179(46.25)	53947.80(9440.23,85607.15)	1983(15.59)	30829.91(9052.86,43976.17)
5-17	65(16.80)	34324.41(3923.06,65078.17)	1407(13.19)	27794.09(16818.74,35466.54)
18-64	50(12.92)	7927.16(5000.42,14573.96)	5192(48.67)	8792.30(4842.59,23840.39)
≥65	2(0.52)	6206.16	941(8.82)	7243.11(4630.49,10952.15)
Payment method				
Medical insurance reimbursement	115(29.72)	16290.80(4425.77,67786.50)	5099(47.80)	7899.12(4060.32,25001.25)
Out-of-pocket	272(70.28)	38698.99(6691.63,79832.64)	5569(52.20)	19167.64(6222.59,35531.53)
Hospital nature				
Public	383(98.97)	34324.41(5890.88,74399.60)	10577(99.15)	11954.40(4687.60,31335.88)
Private	4(1.03)	2308.81(1836.38,2890.01)	91(0.85)	6294.61(4358.90,10473.40)
Hospital level				
Provincial and municipal level	363(93.80)	39984.99(7102.87,77171.50)	8455(79.26)	18278.17(6512.16,34180.90)
District and county level and below	24(6.20)	1621.95(040.19,4964.60)	2213(20.74)	4432.37(2863.81,8677.71)
Hospital type				
General hospital	173(44.70)	22075.96(4783.47,65723.81)	7910(74.15)	11721.42(5390.12,28686.40)
Traditional Chinese Medicine (TCM) hospital	5(1.29)	1621.95(1478.33,4452.56)	500(4.69)	3694.46(2477.04,6061.76)
Maternal and Child Healthcare Hospital	204(52.71)	46781.53(8684.96,87415.94)	1733(16.24)	26081.30(2815.06,48250.08)
Specialized hospital	3(0.78)	1621.95	317(2.97)	15346.29(4988.70,36103.38)
Primary medical and health institution	2(0.52)	1325.50	208(1.95)	24216.29(7261.59,44736.85)
Length of stay (days) [mean(SD)]	18.33(16.38)		9.79(8.64)	
Proportion of medications (%) [mean(SD)]	16.40(12.20)		15.71(15.35)	

Number of comorbidities				
0	141(36.43)	37498.43(4783.47,68001.45)	5297(49.65)	17022.13(4562.44,33381.44)
1	54(13.95)	43083.01(3619.92,72878.70)	1878(17.60)	17564.66(6254.63,31249.64)
2	62(16.02)	33992.49(7142.51,68798.93)	1675(15.70)	7865.73(4066.82,21395.32)
≥3	130(33.59)	24556.55(6946.08,85413.60)	1818(17.04)	8460.91(4579.88,29631.30)
Cardiac procedure				
No	138(35.66)	5135.90(2767.60,9773.05)	5778(54.16)	5913.84(2956.37,11664.93)
Yes	249(64.34)	65723.81(35731.61,97378.30)	4890(45.84)	30580.25(16652.31,43654.64)

Supplemental Table S6: Inpatient costs and ICD-10 codes for CHD types and subtypes in Gansu Province from 2015 to 2020

Types of congenital heart disease	ICD-10	n(q%)	Median (lower quartile, upper quartile) (CNY)
Congenital malformations of cardiac chambers and connections	Q20	79(0.71)	15028.7(4068.67,70490.61)
Common arterial trunk	Q20.0	9(0.08)	3021.03(1851.82,3668.77)
Double outlet right ventricle	Q20.1	19(0.17)	32029.57(4961.17,75343.52)
Discordant ventriculoarterial connection	Q20.3	28(0.25)	10964.58(4260.61,112652.00)
Double inlet ventricle	Q20.4	19(0.17)	23314.14(4666.62,79726.66)
Other congenital malformations of cardiac chambers and connections	Q20.8	3(0.03)	51393.25
Congenital malformation of cardiac chambers and connections, unspecified	Q20.9	1(0.01)	3938.06
Congenital malformations of cardiac septa	Q21	5446(49.26)	22635.64(5273.85,37992.73)
Ventricular septal defect	Q21.0	1852(16.75)	33084.17(11959.40,46948.91)
Atrial septal defect	Q21.1	3225(29.17)	18192.05(3966.34,30285.02)
Atrioventricular septal defect	Q21.2	118(1.07)	41648.95(6746.77,65528.04)
Tetralogy of Fallot	Q21.3	204(1.85)	43861.59(6597.02,70786.30)
Aortopulmonary septal defect	Q21.4	4(0.04)	10651.08(2613.13,45779.64)
Other congenital malformations of cardiac septa	Q21.8	41(0.37)	5689.40(3416.69,9857.08)
Congenital malformation of cardiac septum, unspecified	Q21.9	2(0.02)	4107.98
Congenital malformations of pulmonary and tricuspid valves	Q22	66(0.60)	23234.81(6771.81,71681.29)
Pulmonary valve atresia	Q22.0	21(0.19)	17312.29(5111.66,105696.30)
Congenital pulmonary valve stenosis	Q22.1	18(0.16)	29770.62(22837.53,48598.57)
Other congenital malformations of pulmonary valve	Q22.3	1(0.01)	132628.00
Congenital tricuspid stenosis	Q22.4	8(0.07)	14907.11(5690.06,33576.41)
Ebstein anomaly	Q22.5	1(0.01)	5071.10
Hypoplastic right heart syndrome	Q22.6	8(0.07)	32456.70(3697.64,91130.15)
Other congenital malformations of tricuspid valve	Q22.8	5(0.05)	7191.48(6348.27,22248.20)
Other congenital malformations of tricuspid valve	Q22.9	4(0.04)	47948.68(7800.71,89510.77)
Congenital malformations of aortic and mitral valves	Q23	89(0.81)	9255.08(5176.09,74780.87)
Congenital stenosis of aortic valve	Q23.0	8(0.07)	4757.07(2432.15,60357.83)
Congenital insufficiency of aortic valve	Q23.1	50(0.45)	10385.28(6009.52,74780.87)
Congenital mitral stenosis	Q23.2	6(0.05)	37422.51(7930.69,109591.40)

Congenital mitral insufficiency	Q23.3	2(0.02)	4782.18
Hypoplastic left heart syndrome	Q23.4	2(0.02)	4942.04
Other congenital malformations of aortic and mitral valves	Q23.8	7(0.06)	5463.64(2735.46,41180.55)
Congenital malformation of aortic and mitral valves, unspecified	Q23.9	14(0.13)	27751.25(6073.97,94152.20)
Other congenital malformations of heart	Q24	3922(35.48)	7412.11(4267.72,13261.52)
Dextrocardia	Q24.0	9(0.08)	6976.62(2200.07,10805.58)
Laevocardia	Q24.1	3(0.03)	7990.21
Cor triatriatum	Q24.2	6(0.05)	46015.11(39341.46,64593.97)
Congenital subaortic stenosis	Q24.4	5(0.05)	40501.37(21946.83,47627.70)
Congenital subaortic stenosis	Q24.5	1536(13.89)	7601.41(6151.06,9454.61)
Congenital heart block	Q24.6	29(0.26)	27958.03(6913.92,55825.82)
Other specified congenital malformations of heart	Q24.8	45(0.41)	10358.10(5725.47,20924.95)
Congenital malformation of heart, unspecified	Q24.9	2289(20.71)	6477.24(3283.59,23239.15)
Congenital malformations of great arteries	Q25	1372(12.41)	18967.64(4520.40,31178.06)
Patent ductus arteriosus	Q25.0	1100(9.95)	18513.87(3729.83,27919.32)
Coarctation of aorta	Q25.1	43(0.39)	41392.32(21354.00,88243.05)
Atresia of aorta	Q25.2	3(0.03)	5835.90
Stenosis of aorta	Q25.3	1(0.01)	14372.55
Other congenital malformations of aorta	Q25.4	140(1.27)	49313.81(8007.65,115288.30)
Atresia of pulmonary artery	Q25.5	26(0.24)	6309.08(3014.22,45572.59)
Stenosis of pulmonary artery	Q25.6	42(0.38)	15650.52(4995.93,43646.72)
Other congenital malformations of pulmonary artery	Q25.7	13(0.12)	6944.87(5474.68,12590.24)
Other congenital malformations of great arteries	Q25.8	2(0.02)	8378.48
Congenital malformation of great arteries, unspecified	Q25.9	2(0.02)	3914.96
Congenital malformations of great veins	Q26	81(0.73)	45249.73(9606.16,76649.33)
Persistent left superior vena cava	Q26.1	4(0.04)	5801.20(1638.56,11649.91)
Total anomalous pulmonary venous connection	Q26.2	43(0.39)	71443.56(35752.26,112175.70)
Partial anomalous pulmonary venous connection	Q26.3	16(0.14)	48235.00(13457.44,65073.16)
Anomalous pulmonary venous connection, unspecified	Q26.4	8(0.07)	7828.62(4412.75,16408.59)
Anomalous portal venous connection	Q26.5	1(0.01)	2719.41
Other congenital malformations of great veins	Q26.8	9(0.08)	9616.62(6478.14,18994.13)

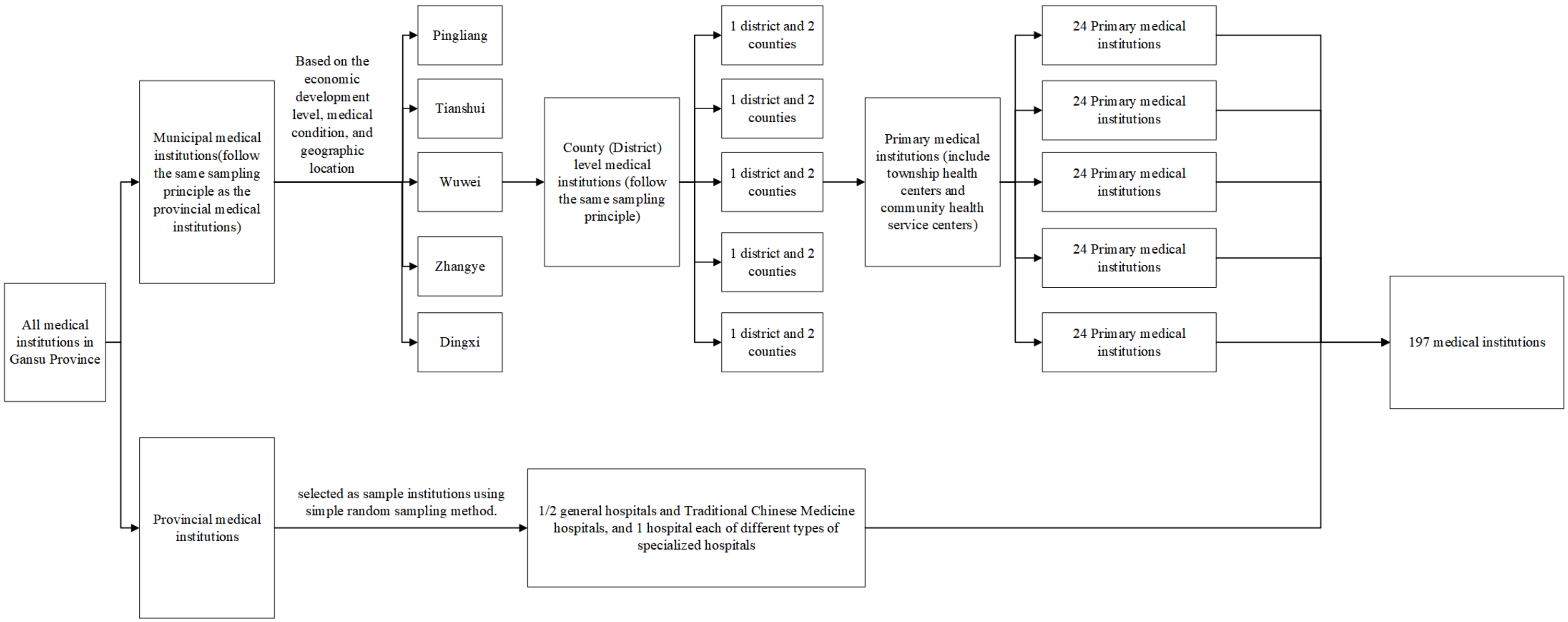

Supplementary Figure S1 Institutional selection process flowchart. Medical institutions were sampled stratified by administrative level. Provincial medical institutions pertain to the administrative level of provinces, municipal medical institutions align with municipal administrative levels, and district (county) medical institutions correspond to the district (county) administrative levels.
